# Development of incisional herniation after midline laparotomy

**DOI:** 10.1002/bjs5.3

**Published:** 2017-05-10

**Authors:** J. J. Harlaar, E. B. Deerenberg, R. S. Dwarkasing, A. M. Kamperman, G. J. Kleinrensink, J. Jeekel, J. F. Lange

**Affiliations:** ^1^ Department of Surgery Erasmus University Medical Centre Rotterdam The Netherlands; ^2^ Department of Radiology Erasmus University Medical Centre Rotterdam The Netherlands; ^3^ Department of Psychiatry Erasmus University Medical Centre Rotterdam The Netherlands; ^4^ Department of Neuroscience Erasmus University Medical Centre Rotterdam The Netherlands

## Abstract

**Background:**

Incisional herniation is a common complication after abdominal surgery associated with considerable morbidity. The aim of this study was to determine whether incisional hernia is an early complication, in order to understand better the aetiology of incisional hernia formation.

**Methods:**

This study involved the secondary analysis of a subset of patients included in a large RCT comparing small and large tissue bites (5 mm every 5 mm, or 1 cm every 1 cm) in patients scheduled to undergo elective abdominal surgery by midline laparotomy. The distance between the rectus abdominis muscles (RAM distance) was measured by standardized ultrasound imaging 1 month and 1 year after surgery. The relationship between the 1‐year incidence of incisional hernia and the RAM distance at 1 month was investigated.

**Results:**

Some 219 patients were investigated, 113 in the small‐bites and 106 in the large‐bites group. At 1 month after surgery the RAM distance was smaller for small bites than for large bites (mean(s.d.) 1·90(1·18) versus 2·39(1·34) cm respectively; P = 0·005). At 1 year, patients with incisional hernia had a longer RAM distance at 1 month than those with no incisional hernia (mean(s.d.) 2·43(1·48) versus 2·03(1·19) cm respectively; relative risk 1·14, 95 per cent c.i. 1·03 to 1·26, P = 0·015).

**Conclusion:**

A RAM distance greater than 2 cm at 1 month after midline laparotomy is associated with incisional hernia. Closure with small bites results in a smaller distance between the muscles.

## Introduction

Despite many decades of research there is little information about the aetiology of incisional hernia formation. Several hypotheses have been proposed to explain the development of these hernias^1^. Surgical technique seems important, and two clinical trials^2^ ^3^ have suggested that an increased distance between the rectus abdominis muscles (RAM distance) 1 month after surgery predicts later incisional hernia formation.

A recent RCT demonstrated that a running suture technique with small tissue bites resulted in a reduced incidence of incisional hernia compared with that after use of a running suture technique with large tissue bites^4^. In that study, small tissue bites were defined as placement of a suture every 5 mm from the wound edge at 5‐mm intervals, based on preclinical studies^5^ ^6^ that suggested small bites induced wound healing, collagen type l formation and higher bursting strength. The question of whether incisional herniation is an early complication, and how the small‐bites technique may reduce its formation, is still unanswered.

The aim of the present study was to determine whether the RAM distance 1 month after surgery could predict incisional hernia formation, and whether this distance was related to the small‐bites technique.

## Methods

This study comprises an explanatory secondary analysis of the STITCH (Suture Techniques to reduce the Incidence of The inCisional Hernia) trial, a prospective, multicentre, double‐blinded RCT of patients scheduled for elective abdominal operation through a midline incision (http://ClinicalTrials.gov registration number NCT01132209; Netherlands Trial Register NTR2052). The trial protocol and primary endpoint results have been published previously^4^ ^7^. Patients aged 18 years or more were asked to participate in the trial at the outpatient clinic or in hospital on the day before surgery. Patients with a history of incisional hernia or fascial dehiscence after midline laparotomy, abdominal surgery through a midline incision within the previous 3 months, current pregnancy or participation in another intervention trial were excluded.

The study protocol was approved by the institutional review board (IRB) of Erasmus University Medical Centre (Erasmus MC), Rotterdam (MEC‐2009‐026), and by the IRBs of each study centre before the start of inclusion. All participants gave written informed consent. An independent data and safety monitoring board (DSMB), consisting of two independent surgeons and one biomedical statistician, was constituted before the start of the trial. All serious adverse events, defined as death and burst abdomen, that occurred during the study were reported to the IRB of Erasmus MC. The progress of the trial and all adverse events were reported every 3 months to the DSMB, and the safety of the trial was examined. The DSMB had access to unblinded data whenever deemed necessary.

Patients were assigned randomly to closure with large tissue bites or with small tissue bites. In the intervention group, the principle of the small tissue bites technique consisted of placing at least twice as many stitches as the incision length in centimetres with USP 2/0 PDS Plus II™ (Ethicon, Somerville, New Jersey, USA) with a 31‐mm needle^7–10^. The suture technique was applied with tissue bites of 5 mm and intersuture spacing of 5 mm. In the control group, the conventional large tissue bites or mass closure technique was applied with tissue bites of at least 1 cm and intersuture spacing of 1 cm with USP 1 double loop PDS Plus II™ (Ethicon) with a 48‐mm needle.

The primary outcome was the occurrence of incisional hernia in the laparotomy scar and the RAM distance at 1 month and 1 year after surgery. All patients who completed both of these examinations were included in the study. Patients who had a relaparotomy within 1 year were excluded from analysis, to prevent the effect of several closure techniques in the outcome analysis.

**Figure 1:**
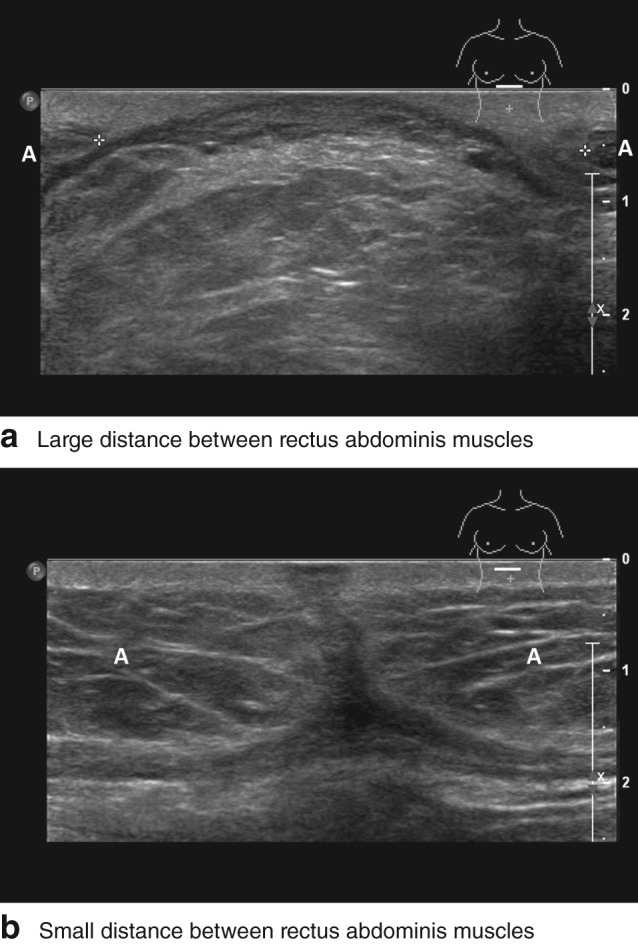
Ultrasound images at 1 month after surgery from a 63‐year‐old man with a median laparotomy scar from xiphoid to umbilicus. **a** At the upper one‐third level of the laparotomy scar, bulging of intra‐abdominal fatty tissue through a large distance of 4·3 cm between the medial borders (indicated by + markers) of the rectus abdominis muscles can be seen. The patient developed an incisional hernia during follow‐up. **b** At the two‐thirds level of the laparotomy scar, a tight junction is visible between the medial borders of the rectus abdominis muscles in the midline. **a,b** The body mark (upper left) indicates the level and position (axially oriented) of the ultrasound probe (10–12 MHz, linear array transducer). A, rectus abdominis muscle

Patients were invited for follow‐up at the outpatient clinic 1 month and 1 year after surgery, when they underwent physical examination by a medical doctor and abdominal ultrasound imaging by a radiologist, both blinded to the intervention group. Ultrasound examinations were performed in a standard fashion with a focus on the RAM distance and the occurrence of incisional hernia in the laparotomy scar at 1 month and 1 year after surgery (*Fig*. 1). After ultrasonographic examination of the entire scar, RAM distance was measured at three levels: the cranial upper one‐third of the entire incision, the caudal lower one‐third, and the maximum RAM distance. For further analysis, the maximum distance was used. The European Hernia Society definition of incisional hernia was used: ‘any abdominal wall gap with or without bulge in the area of a postoperative scar perceptible or palpable by clinical examination or imaging’^11^.

### Statistical analysis

Differences between randomized groups were analysed with *t* tests for continuous variables and χ^2^ tests for categorical variables. The 1‐year incidence of incisional hernia and its relationship with the RAM distance at 1 month was evaluated. The primary outcome was analysed by logistic regression analysis. Multivariable logistic regression analysis was used to adjust for confounders^12^. A co‐variable was deemed a confounding variable when it showed a significant relationship with both the RAM distance at 1 month and the presence of incisional hernia in the univariable regression analysis. Relative risks (RRs) with 95 per cent confidence intervals of the adjusted and unadjusted analysis are reported^13^. Relationships between suture characteristics and RAM at 1 month were determined by means of Pearson correlations.

The baseline co‐variables considered were the following predefined, potential confounders for incisional hernia development: abdominal aortic aneurysm, BMI, diabetes mellitus, corticosteroid use, preoperative chemotherapy, preoperative radiotherapy, chronic obstructive pulmonary disease (COPD), smoking, age, collagen disorders, non‐incisional hernias (including inguinal hernia) and cardiovascular disease^7^. Statistical analysis was performed with IBM SPSS^®^ version 20.0 software (IBM, Armonk, New York, USA).

## Results

**Figure 2:**
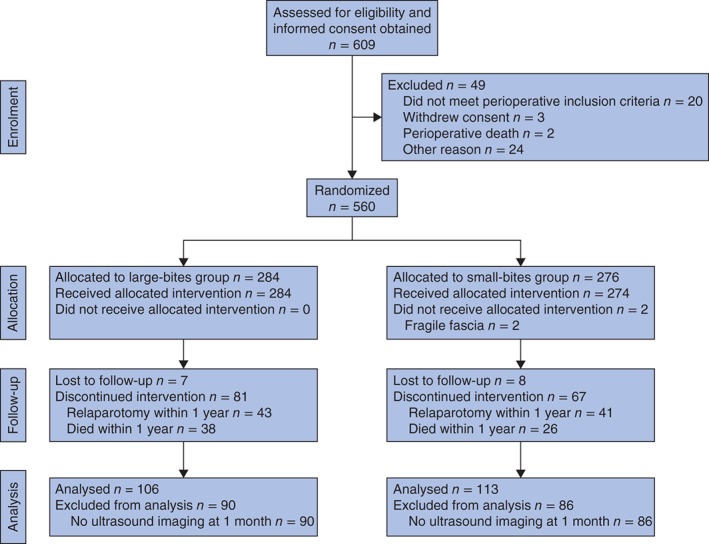
CONSORT flow diagram for the study^14^

Between October 2009 and March 2012, 219 patients (113 with small bites, 106 with large bites) from a total of 560 completed the standard ultrasound examination 1 month and 1 year after surgery. Patients who had relaparotomy within 1 year were excluded from the analysis (*Fig*. 2). Follow‐up ended in August 2013.

Baseline characteristics were similar for the two groups except in relation to COPD, smoking and corticosteroid use, for which the proportion was significantly higher in the small‐bites group (*Table* 1). Most operations were resections undertaken for gastrointestinal neoplasms. *Table* 2 shows details of the suture techniques employed. Incisional herniation was identified in 38 of 106 patients (35·8 per cent) in the large‐bites group and 22 of 113 patients (19·5 per cent) in the small‐bites group (RR 1·56, 95 per cent c.i. 1·09 to 2·23; *P* = 0·007). Eighty (36·5 per cent) of the 219 patients had postoperative complications, the incidence of which did not differ significantly between the groups (*Table* 3).

**Table 1:** Baseline characteristics

	Large bites (*n* = 106)	Small bites (*n* = 113)	*P* ‡
Age (years)*	62·4(12·6)	61·8(14·3)	0·723§
Sex ratio (M : F)	55 : 51	49 : 64	0·207
BMI (kg/m^2^)*	25·5(4·5)	25·4(4·4)	0·860§
Smoking	17 (16·0)	33 (29·2)	0·020
Diabetes mellitus	11 (10·4)	9 (8·0)	0·536
COPD	9 (8·5)	20 (17·7)	0·047
Cardiovascular disease	40 (37·7)	43 (38·1)	0·961
Corticosteroid usage	1 (0·9)	10 (8·8)	< 0·001
Non‐incisional hernia†	12 (11·3)	16 (14·2)	0·530
AAA	3 (2·8)	5 (4·4)	0·530
Previous laparotomy	19 (17·9)	21 (18·6)	0·900
ASA fitness grade			0·876
I	24 (22·6)	26 (23·0)	
II	64 (60·4)	65 (57·5)	
≥ III	18 (17·0)	22 (19·5)	
Preoperative chemotherapy	20 (18·9)	22 (19·5)	0·910
Preoperative radiotherapy	16 (15·1)	26 (23·0)	0·137
Type of surgery			0·723
Gynaecological	12 (11·3)	18 (15·9)	
Upper gastrointestinal	22 (20·8)	19 (16·8)	
Lower gastrointestinal	61 (57·5)	65 (57·5)	
Vascular	11 (10·4)	11 (9·7)	

Values in parentheses are percentages unless indicated otherwise;

* values are mean(s.d.).

† History of non‐incisional hernia (for example inguinal, umbilical or epigastric hernia). COPD, chronic obstructive pulmonary disease; AAA, abdominal aortic aneurysm.

‡ χ^2^ test, except

§ *t* test.

**Table 2:** Details of suture techniques

	Large bites (*n* = 106)	Small bites (*n* = 113)	*P* *
No. of stitches	24·3(6·7)	43·4(12·1)	< 0·001
Total length of used sutures (cm)	94·4(38·4)	107·3(39·7)	0·016
Wound length (cm)	21·6(5·0)	21·7(5·1)	0·851
Suture length to wound length ratio	4·4(1·6)	4·9(1·2)	0·011
Time for fascial closure (min)	9·8(3·4)	13·8(5·5)	< 0·001
No. of sutures to wound length ratio	1·1(0·3)	2·0(0·4)	< 0·001
Suture length to no. of stitches ratio	4·6(5·6)	3·3(6·6)	0·111

Values are mean(s.d.).

* *t* test.

**Table 3:** Incisional hernia and postoperative complications

	Large bites (*n* = 106)	Small bites (*n* = 113)	*P* †
Incisional hernia	38 (35·8)	22 (19·5)	0·007
Patients with postoperative complications	37 (34·9)	43 (38·1)	0·629
Ileus	7 (6·6)	13 (11·5)	0·208
Pneumonia	10 (9·4)	8 (7·1)	0·526
Cardiac event	9 (8·5)	4 (3·5)	0·121
Surgical‐site infection*	23 (21·7)	17 (15·0)	0·203

Values in parentheses are percentages.

* Detailed criteria for surgical‐site infections can be found in the published study protocol^7^.

† χ^2^ test.

At 1 month after surgery the RAM distance was less in patients who had small bites (mean(s.d.) 1·90(1·18) (range 0·10–9·10) cm) than in those with large bites (2·39(1·34) (0·20–7·00) cm) (*P* = 0·005). At 1 year the distance had increased in both groups, but remained less in the small‐bites group (2·76(1·41) (0·10–9·00) cm *versus* 3·32(2·06) (0·10–6·00) cm in the large‐bites group; *P* = 0·031).

Patients with an incisional hernia had a greater RAM distance at 1 month compared with those without an incisional hernia at the 1‐year follow‐up (mean(s.d.) 2·43(1·48) *versus* 2·03(1·19) cm respectively). There was a linear correlation between an enlarged RAM distance at 1 month and the likelihood of incisional hernia at 1 year of 14 per cent per centimetre of widening (unadjusted RR 1·14, 95 per cent c.i. 1·03 to 1·26; *P* = 0·015). A distance of 2 cm or more at 1 month after surgery increased the risk of developing an incisional hernia by 32 per cent (unadjusted RR 1·32, 0·94 to 1·86; *P* = 0·090). Age of the patient, BMI and the presence of cardiovascular disease were shown to confound the relationship between RAM distance at 1 month and the risk of incisional hernia at 1 year. Adjustment of the relationship for these confounders marginally lowered the incremental risk to 12 per cent per centimetre of widening (adjusted RR 1·12, 0·99 to 1·27; *P* = 0·085).

The Pearson test showed a significant correlation between the RAM distance and closure time (*r* = −0·06, *P* = 0·030).

## Discussion

This study has confirmed that incisional hernia develops as an early complication after abdominal surgery. Compared with large bites, the small‐bites suture technique resulted in a smaller RAM distance, which was associated with a lower incidence of incisional hernia. This finding confirms the hypothesis that the small‐bites suture technique would result in less separation of the fascial edges.

A linear correlation was found between an enlarged RAM distance at 1 month and the likelihood of incisional herniation being present at 1 year of 14 per cent per centimetre of widening. In the present study, a RAM distance above 20 mm appeared to be the cut‐off point, although earlier studies^2^ ^3^ suggested that 12‐mm and 15‐mm separation of the fascial edges or RAM distance represent cut‐off points for risk of incisional hernia formation. These differences may be caused by differences in methodology of radiological examination, although it has been shown^15^ that a RAM distance of 20 mm at the level of the umbilicus is normal in an unoperated population.

Ultrasonography offers the advantages of real‐time imaging with no exposure to ionizing radiation, but is investigator‐dependent. Risk of bias in the present study was minimized by blinding the radiologist, using standardized outcomes and objective measurements. Earlier studies used CT or metal clips and X‐ray examination, but for the present study it was considered that exposing patients to unnecessary radiation was unacceptable.

Preclinical studies^5^ ^16^ have shown that small tissue bites prevent separation of the fascial edges in the early postoperative phase. The present study also shows that small bites provide better conditions for fascial healing, possibly due to avoidance of necrosis of the rectus abdominis muscles and improved distribution of forces. There was a significant negative correlation between closure time and RAM distance at 1 month after surgery, reflecting the longer time taken for closure with the small‐bites technique. This investment in time, however, did result in fewer incisional hernias.

This study has limitations. Despite 560 patients being randomized, it was difficult to schedule patients for the standardized ultrasound imaging at 1 month. Patients who had a relaparotomy, those who died within 1 year of follow‐up, and patients who did not have ultrasound imaging at 1 month or 1 year could not be analysed. This selection led to a high incidence of patients with an incisional hernia. There were significantly more patients with COPD, corticosteroid use and tobacco use in the small‐bites group. In the adjusted analyses, age, BMI and presence of cardiovascular disease were confounders in the relationship between RAM distance at 1 month and the risk of incisional herniation at 1 year. These are known risk factors for incisional hernia formation and may have influenced the wound‐healing process^17^.

RAM distance increased with time, independent of the suture technique employed. From earlier studies it is known that the rate of incisional hernia increases during longer follow‐up^18^. In a comparison of suture repair with mesh repair of incisional hernia, delayed incisional hernia recurrence was shown after 10 years of follow‐up^19^. Experimental evidence, however, is supportive of the small‐bites technique. A suture technique with an equal distribution of forces on the fascia is necessary to achieve an optimal collagen I/III ratio. Too great a tensile force per suture results in more scar tissue^6^ ^16^. The holding force of a suture depends on the collagen that is deposited in the suture, best achieved by suturing the aponeurosis without muscle or fat tissue^20^. Long‐term follow‐up studies will show whether the protective effect of small bites can be maintained.
